# Novel lipoarabinomannan point-of-care tuberculosis test for people with HIV: a diagnostic accuracy study

**DOI:** 10.1016/S1473-3099(19)30001-5

**Published:** 2019-08

**Authors:** Tobias Broger, Bianca Sossen, Elloise du Toit, Andrew D Kerkhoff, Charlotte Schutz, Elena Ivanova Reipold, Amy Ward, David A Barr, Aurélien Macé, Andre Trollip, Rosie Burton, Stefano Ongarello, Abraham Pinter, Todd L Lowary, Catharina Boehme, Mark P Nicol, Graeme Meintjes, Claudia M Denkinger

**Affiliations:** a FIND, Geneva, Switzerland; b Department of Medicine, Faculty of Health Sciences, University of Cape Town, Cape Town, South Africa; c Wellcome Center for Infectious Diseases Research in Africa, Institute of Infectious Disease and Molecular Medicine, University of Cape Town, Cape Town, South Africa; d Division of Medical Microbiology, University of Cape Town, Cape Town, South Africa; e National Health Laboratory Service, Cape Town, South Africa; f Division of HIV, Infectious Diseases and Global Medicine, Zuckerberg San Francisco General Hospital, University of California, San Francisco, San Francisco, CA, USA; g Wellcome Trust Liverpool Glasgow Centre for Global Health Research, University of Liverpool, Liverpool, UK; h FIND, Cape Town, South Africa; i Southern African Medical Unit, Médecins sans Frontières, Cape Town, South Africa; j Public Health Research Institute Center, New Jersey Medical School, Rutgers University, Newark, NJ, USA; k Department of Chemistry and Alberta Glycomics Centre, University of Alberta, Edmonton, AB, Canada

## Abstract

**Background:**

Most tuberculosis-related deaths in people with HIV could be prevented with earlier diagnosis and treatment. The only commercially available tuberculosis point-of-care test (Alere Determine TB LAM Ag [AlereLAM]) has suboptimal sensitivity, which restricts its use in clinical practice. The novel Fujifilm SILVAMP TB LAM (FujiLAM) assay has been developed to improve the sensitivity of AlereLAM. We assessed the diagnostic accuracy of the FujiLAM assay for the detection of tuberculosis in hospital inpatients with HIV compared with the AlereLAM assay.

**Methods:**

For this diagnostic accuracy study, we assessed biobanked urine samples obtained from the FIND Specimen Bank and the University of Cape Town Biobank, which had been collected from hospital inpatients (aged ≥18 years) with HIV during three independent prospective cohort studies done at two South African hospitals. Urine samples were tested using FujiLAM and AlereLAM assays. The conduct and reporting of each test was done blind to other test results. The primary objective was to assess the diagnostic accuracy of FujiLAM compared with AlereLAM, against microbiological and composite reference standards (including clinical diagnoses).

**Findings:**

Between April 18, 2018, and May 3, 2018, urine samples from 968 hospital inpatients with HIV were evaluated. The prevalence of microbiologically-confirmed tuberculosis was 62% and the median CD4 count was 86 cells per μL. Using the microbiological reference standard, the estimated sensitivity of FujiLAM was 70·4% (95% CI 53·0 to 83·1) compared with 42·3% (31·7 to 51·8) for AlereLAM (difference 28·1%) and the estimated specificity of FujiLAM was 90·8% (86·0 to 94·4) and 95·0% (87·7–98·8) for AlereLAM (difference −4·2%). Against the composite reference standard, the specificity of both assays was higher (95·7% [92·0 to 98·0] for FujiLAM *vs* 98·2% [95·7 to 99·6] for AlereLAM; difference −2·5%), but the sensitivity of both assays was lower (64·9% [50·1 to 76·7] for FujiLAM *vs* 38·2% [28·1 to 47·3] for AlereLAM; difference 26·7%).

**Interpretation:**

In comparison to AlereLAM, FujiLAM offers superior diagnostic sensitivity, while maintaining specificity, and could transform rapid point-of-care tuberculosis diagnosis for hospital inpatients with HIV. The applicability of FujiLAM for settings of intended use requires prospective assessment.

**Funding:**

Global Health Innovative Technology Fund, UK Department for International Development, Dutch Ministry of Foreign Affairs, Bill & Melinda Gates Foundation, German Federal Ministry of Education and Research, Australian Department of Foreign Affairs and Trade, Wellcome Trust, Department of Science and Technology and National Research Foundation of South Africa, and South African Medical Research Council.

## Introduction

Tuberculosis is the leading infectious cause of death globally and remains the most common cause of mortality in people with HIV, causing an estimated 300 000 deaths in 2017.^1^ Most tuberculosis-related deaths in people with HIV could be prevented with earlier diagnosis and treatment.^1^ Extrapulmonary tuberculosis is more common in people with HIV who are severely immunocompromised than in immunocompetent people and therefore sputum might not represent the ideal diagnostic sample.^2^ Furthermore, independent of location of disease, producing a sputum sample is often difficult for patients with advanced HIV who are severely ill. As a result, non-sputum-based tests have been identified as an urgent unmet clinical need by WHO.^3^

The Alere Determine TB LAM Ag assay (AlereLAM; Abbott, Chicago, IL, USA) detects the presence of the mycobacterial cell wall component, lipoarabinomannan (LAM), in a urine sample. However, in a meta-analysis by WHO,^4^, ^5^ the sensitivity of this test was only 45% in people with HIV, with higher sensitivity (56%) in patients with CD4 counts equal to or less than 100 cells per μL. Despite suboptimal sensitivity, the test reduces mortality when implemented for immunocompromised hospital inpatients with HIV.^6^ On this basis, WHO recommends the use of AlereLAM for people with HIV and a CD4 count equal to or less than 100 cells per μL and in those defined as seriously ill according to WHO criteria (respiratory rate >30 breaths per min, body temperature >39°C, heart rate >120 beats per min, or unable to walk unaided).^4^ A more sensitive, rapid urine-based test could widen the indication for testing and improve the diagnosis of tuberculosis and associated outcomes in people with HIV.^3^

**Research in context**

**Evidence before this study**

WHO recommended the use of the rapid, point-of-care Alere Determine TB LAM Ag assay (AlereLAM) for the diagnosis of tuberculosis in people with HIV. This recommendation was informed by a Cochrane systematic review and meta-analysis of 12 cross-sectional or cohort studies that showed a relatively low pooled sensitivity of 45% (95% CI 29–63) and specificity of 92% (80–97) against a microbiological reference standard. In a subgroup of patients with HIV and CD4 counts less than or equal to 100 cells per μL, pooled sensitivity was 56% (41–70).

We searched PubMed for articles published between Feb 5, 2015, and Sept 17, 2018, evaluating the diagnostic accuracy or diagnostic yield of AlereLAM using the search terms (tuberculosis or TB) AND (lipoarabinomannan or LAM) AND (test OR assay OR antigen OR Ag OR lateral flow assay* OR urine antigen OR point of care) AND (accuracy OR sensitivity OR specificity OR yield OR diagnos* OR screening). Our search yielded an additional 23 relevant studies, which confirmed the moderate clinical sensitivity of AlereLAM. The search also identified two randomised trials that demonstrated reduced mortality with AlereLAM point-of-care testing for tuberculosis among severely ill inpatients with HIV.

The novel urine-based Fujifilm SILVAMP TB LAM (FujiLAM) assay was developed to overcome the limited sensitivity of AlereLAM and increase the diagnostic yield of rapid urinary lipoarabinomannan testing. On Sept 17, 2018, we did a second PubMed search using the search term (tuberculosis or TB) AND (lipoarabinomannan or LAM) AND (Fuji*), but no articles were identified.

**Added value of this study**

This is the first study to assess the accuracy and diagnostic yield of the FujiLAM assay for the diagnosis of active tuberculosis. Diagnostic accuracy was compared with rigorously defined microbiological and composite reference standards in three cohorts of hospital inpatients with HIV. The findings from this study show that FujiLAM is substantially more sensitive than AlereLAM, while maintaining specificity, for the diagnosis of active tuberculosis in hospital inpatients with HIV.

**Implications of all the available evidence**

Considering the substantially improved sensitivity of FujiLAM compared with AlereLAM and the high diagnostic yield compared with sputum-based diagnostics, the FujiLAM assay has the potential to substantially improve rapid diagnosis of tuberculosis in patients with HIV who are admitted to hospital and potentially people with HIV in the general population. Since AlereLAM has demonstrated survival benefit, FujiLAM might potentially further reduce tuberculosis-related mortality in people with HIV. These findings will inform a WHO policy review for lipoarabinomannan-based diagnostic tests of active tuberculosis. Further research, including prospective and operational studies on the FujiLAM assay in settings of intended use and in additional patient populations, including outpatients with HIV, populations without HIV, and paediatric populations, are needed.

A novel urine-based assay, Fujifilm SILVAMP TB LAM (FujiLAM; Fujifilm, Tokyo, Japan), has been developed that also detects lipoarabinomannan on an instrument-free platform, with results available in less than 1 h. This assay combines a pair of high affinity monoclonal antibodies directed towards largely *Mycobacterium tuberculosis*-specific lipoarabinomannan epitopes^7^, ^8^, ^9^ and a silver-amplification step^10^ that increases the visibility of test and control lines. This enables the detection of urinary lipoarabinomannan concentrations that are approximately 30 times lower than that detected by AlereLAM and improved analytical specificity compared with AlereLAM, which in contrast, uses conventional lateral flow immunoassay technology and polyclonal antibodies.^7^

In this study, we aimed to assess the diagnostic accuracy of FujiLAM for the detection of active tuberculosis compared with AlereLAM in three cohorts of hospital inpatients with HIV, in whom the AlereLAM assay is recommended for use.

## Methods

### Study participants

In this diagnostic accuracy study, we assessed urine samples from the FIND Specimen Bank and the University of Cape Town Biobank obtained from inpatients (aged ≥18 years) with HIV, collected in three independent prospective cohort studies (two unpublished and one published^2^, ^11^) done at two district hospitals in South Africa (appendix). These cohorts were selected for inclusion on the basis of the availability of frozen urine samples for a full cohort of hospital inpatients with HIV in tuberculosis endemic settings, in whom a comprehensive work-up was done to identify tuberculosis or alternative diagnoses. Standard national guidelines^12^ for tuberculosis and HIV management were used across all three cohorts. Cohort 1 included adults with tuberculosis symptoms who were able to produce sputum, and were enrolled regardless of CD4 count on admission to Khayelitsha Hospital (Cape Town, South Africa) between Feb 22, 2017, and August 31, 2017. Patients with extrapulmonary disease without respiratory symptoms were excluded. Cohort 2 included adults with HIV who were admitted to medical wards at GF Jooste Hospital (Cape Town, South Africa) between June 6, 2012, and Oct 4, 2013, regardless of CD4 count, their ability to produce sputum, or whether or not they reported tuberculosis symptoms.^2^, ^11^ Study staff systematically attempted to collect urine, blood, and two sputum samples for testing within 24 h of hospital admission. Cohort 3 included adults with HIV with a CD4 count equal to or less than 350 cells per μL in whom tuberculosis was considered the most likely diagnosis at presentation, who were admitted to Khayelitsha Hospital between Jan 16, 2014, and Oct 19, 2016. All cohorts excluded patients who were already receiving tuberculosis therapy (appendix). In cohorts 1 and 2, enrolment was done consecutively. In cohort 3, patients were randomly enrolled using a dice on a daily basis after all potentially eligible patients were identified. In all cohorts, patients were enrolled on admission to hospital. Sputum, blood, and urine specimens for *M tuberculosis* reference standard testing were collected at enrolment and additional clinical samples were obtained during hospital admission and at follow-up. Follow-up was 8 weeks for cohort 1, and 12 weeks for cohorts 2 and 3 (appendix).

All studies were approved by the Human Research Ethics Committee of the University of Cape Town (Cape Town, South Africa). Written informed consent was obtained from patients, as per study protocols. Study participation did not affect standard of care. This study is reported in accordance with the Standards for Reporting of Diagnostic Accuracy Studies guidelines.^13^ Retrospective urine lipoarabinomannan testing was supervised by the study sponsor (FIND, Geneva, Switzerland) and was done at the University of Cape Town.

### Procedures

Frozen urine aliquots of unprocessed urine were thawed to ambient temperature and mixed manually. Samples that were not immediately used for testing were stored at 4°C for a maximum of 4 h.

**Figure 1:**
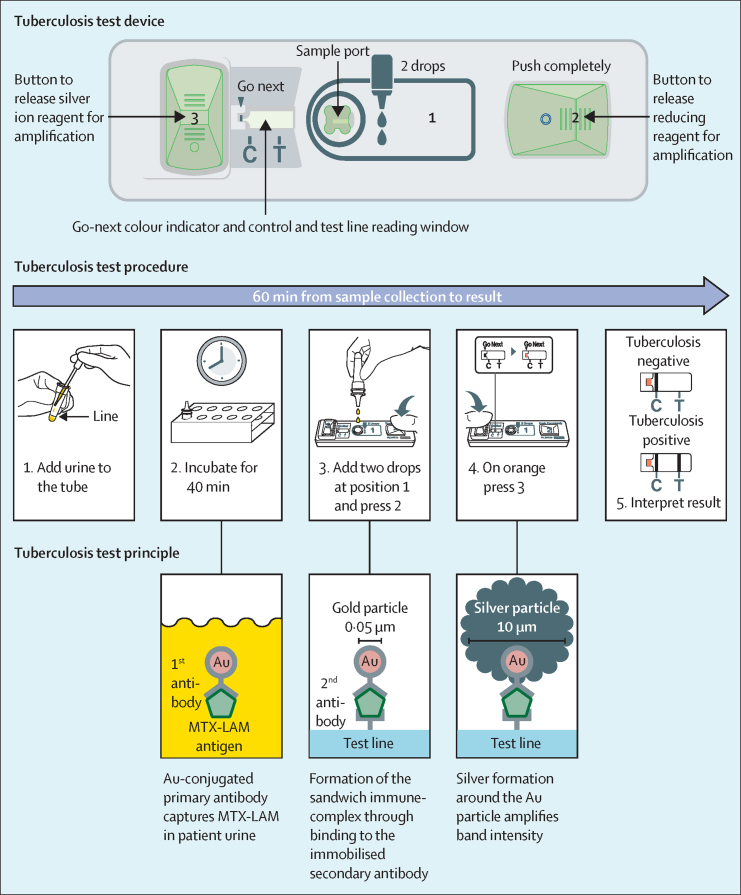
Fujifilm SILVAMP TB LAM test device, procedure, and principle One antibody binds to tetra-arabinoside and hexa-arabinoside structures in the arabinan domain of lipoarabinomannan and the other antibody targets MTX-Man capping motifs of lipoarabinomannan (MTX-Man refers to mannose caps further modified with a 5-methylthio-D-xylofuranose residue).^7^, ^8^, ^9^ Au=gold. C=control line. MTX-LAM=5-methylthio-D-xylofuranose-lipoarabinomannan. T=test line.

AlereLAM testing was done according to the manufacturer's instructions. Briefly, 60 μL urine was applied to the sample pad. After 25 min, test strips were read using the test's reference scale card for grading. In parallel, testing with the FujiLAM was done according to manufacturer's instructions using urine from the same aliquots. The five-step test procedure (figure 1; video) took 50–60 min from sample collection to result. Briefly, urine was added to the reagent tube up to the indicator line (approximately 200 μL), mixed, and incubated for 40 min at ambient temperature. After mixing again, two drops of urine were added to the test strip. Following this, button two was immediately pressed to release a reducing agent for silver amplification. After the Go Next colour indicator mark turned orange (within 3–10 min), button 3 was pressed to release a silver-ion solution to activate the silver amplification reaction. The result was read within 10 min. The FujiLAM assay does not use a reference scale card and any line identified on the test line was deemed positive.

Both AlereLAM and FujiLAM were independently read by two readers masked to the test results of the index or comparator test, respectively, patient status, and all other test results. After interpretation of the initial independent test, readers compared results and, in case of discordance, re-inspected the test strip to establish the final consensus result (through mutual agreement) that was used for analysis. In the case of FujiLAM or AlereLAM assay failure, the test was repeated once.

For reference standard testing, the specimens were processed using standardised protocols at centralised accredited laboratories of the South African National Health Laboratory Service (Cape Town, South Africa). The number of samples tested and testing flow for each cohort are shown in the appendix.

Sputum collection in all cohorts was done by an experienced nurse or trained clinical research worker and sputum induction was done, when required, as described previously.^11^ Reference standard testing was done on all available sputum specimens and included Xpert MTB/RIF (Xpert; Cepheid, Sunnyvale, USA; testing pre-dated rollout of Xpert Ultra MTB/RIF), smear fluorescence microscopy after auramine O staining, mycobacteria growth indicator tube liquid culture (Becton Dickinson, Franklin Lakes, NJ, USA) and solid culture on Löwenstein-Jensen medium. The presence of *M tuberculosis* complex in solid and liquid culture was confirmed with MPT64 antigen detection or MTBDRplus line probe assays (Hain Lifesciences, Nehren, Germany). Blood cultures from all participants were done in BACTEC Myco/F Lytic culture vials (Becton Dickinson) and WHO prequalified in-vitro diagnostic tests were used for HIV testing (rapid diagnostic tests) and CD4 cell counting (flow cytometry). For urinary Xpert testing, 20–40 mL urine was centrifuged and following removal of supernatant the pellet was re-suspended in the residual urine volume and a 0·75 mL sample was used for testing.^2^ For cohorts 2 and 3, additional respiratory and non-respiratory samples such as pleural fluid, cerebrospinal fluid, and tissue fine needle aspirates were obtained, when clinically indicated, and tested using MGIT culture or Xpert. Clinical information and FujiLAM and AlereLAM results were not available to the assessors of the reference standard at the time of testing.

Before data analysis, clinical investigators, who were masked to index test results, categorised patients as having definite tuberculosis, possible tuberculosis, not tuberculosis, and unclassifiable using a combination of clinical and laboratory findings (appendix). Patients with definite tuberculosis had microbiologically confirmed *M tuberculosis* (any culture or any Xpert positive result for *M tuberculosis* during admission). Patients defined as not-tuberculosis had negative microscopy, cultures, and Xpert test results for *M tuberculosis* (and at least one non-contaminated negative culture result), had not started tuberculosis treatment, and were alive or had improvement in clinical tuberculosis symptoms at 3 months' follow-up. Patients defined as possible tuberculosis did not satisfy the criteria for definite tuberculosis, but had clinical or radiological features suggestive of tuberculosis and were started on tuberculosis treatment. Patients that did not fall into any of these categories were defined as unclassifiable and were removed from the main analyses (appendix).

### Statistical analysis

For the primary analysis, we calculated the point estimates and 95% CIs for the sensitivity, specificity, positive predictive value, negative predictive value, positive likelihood ratio, and negative likelihood ratios of FujiLAM and AlereLAM assays by comparison with a microbiological reference standard and a composite reference standard. Definite tuberculosis versus not-tuberculosis diagnostic classifications were used to allocate patients into reference standard positive versus reference standard negative groups. The possible tuberculosis group was deemed negative within a microbiological reference standard but positive within a composite reference standard. Diagnostic accuracy was determined separately for each cohort as per protocol. In a sensitivity analysis, unclassifiable patients were included to assess the effect of exclusions on diagnostic accuracy (appendix). Heterogeneity between cohorts was assessed using Cochran's Q test (appendix).^15^

We did a post-hoc analysis to estimate pooled sensitivity and specificity across cohorts and CD4 strata, using a Bayesian bivariate random-effects model to account for differences in study design.^16^ Simple pooling estimates, as planned a priori, are presented in the appendix with 95% CIs based on Wilson's score method.^17^ The 95% CIs of the sensitivity and specificity differences of the three cohorts for FujiLAM compared with AlereLAM was computed using Tango's score method.^18^ The difference between two tests was considered to be significant if the 95% CIs did not overlap. Cohen's κ statistic^19^ was used to calculate agreement of positive and negative results between the two independent readers of the lipoarabinomannan tests.

In an additional post-hoc analysis, we used the total number of microbiologically confirmed tuberculosis patients, (defined as the detection of *M tuberculosis* by culture or Xpert in at least one clinical specimen of any type) to calculate the comparative diagnostic yield of a single FujiLAM, AlereLAM, sputum Xpert (version G4), urine Xpert, and sputum smear microscopy test from samples collected within the first 24 h of presentation (appendix). This analysis only included patient samples from cohort 2, because the systematic collection of blood, urine, and two sputum diagnostic samples was attempted whenever possible in all patients in this cohort within the first 24 h of admission.

The study protocol and statistical analysis plan are available in the appendix. All data analysis was done using R (version 3.5.1) and Matlab (version 2017b).

### Role of the funding source

The funders of the study had no role in study design, data collection, data analysis, data interpretation, or writing of the manuscript. The corresponding author had full access to all the data in the study and had final responsibility for the decision to submit for publication.

## Results

**Figure 2:**
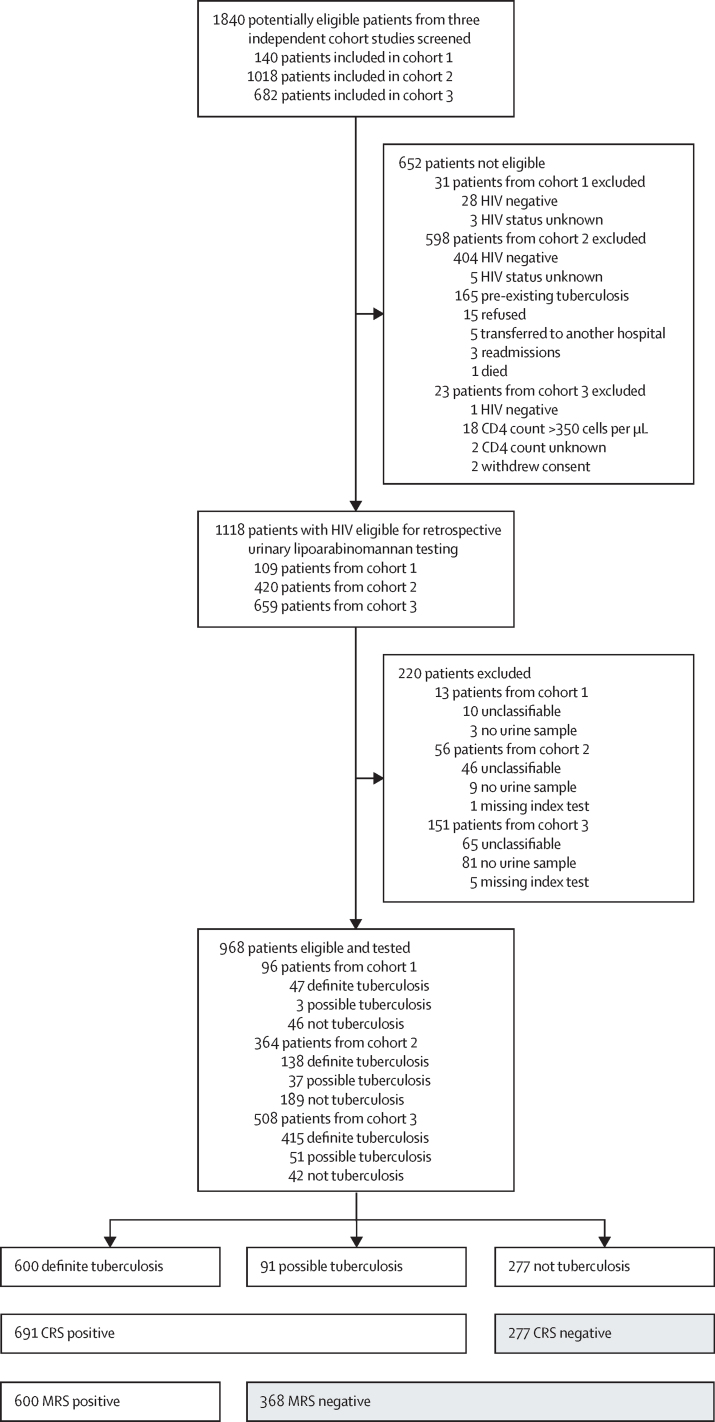
Study flow diagram Details of patients are provided in the appendix. CRS=composite reference standard. MRS=microbiological reference standard.

We evaluated urine samples between April 18, 2018, and May 3, 2018. Of the 1840 patients included in the three independent cohort studies, 1188 patients were eligible for retrospective urinary lipoarabinomannan testing. Of the 1188 eligible patients, 220 patients were excluded from the main analysis because of unavailability of a urine sample (n=93), failed FujiLAM tests (n=6), or unclassifiable diagnostic status (n=121; figure 2). The primary reasons for which patients were deemed unclassifiable were death before diagnosis (n=62) and loss to follow-up where a vital status or an improvement in clinical status was required for diagnosis (n=17; appendix). Of the 968 patients included in the main analysis (96 patients from cohort 1, 364 patients from cohort 2, and 508 patients from cohort 3), 600 (62%) were classified as definite tuberculosis, 91 (9%) as possible tuberculosis, and 277 (29%) as not-tuberculosis (table; appendix). The microbiological reference standard for tuberculosis diagnosis was based on a total of 6397 culture and Xpert tests (mean 6·2 tests per patient) and included 3261 tests on sputum samples and 3136 tests on non-sputum samples (appendix). 236 (24%) of 968 patients could not provide a sputum sample. Definite tuberculosis diagnosis was based on the results from non-sputum samples for 117 (20%) of 600 patients. Most patients were young immunocompromised adults (median age 35 years [IQR 30–42]), with a median CD4 count of 113 cells per μL (IQR 40–262) in cohort 1, 153 cells per μL (53–313) in cohort 2, and 59 cells per μL (23–122) in cohort 3. 439 (45%) of 968 patients had a history of previous tuberculosis treatment and all patients in cohort 1 and 3 and 329 (90%) of 364 patients in cohort 2 had a positive WHO symptom screen for tuberculosis.

**Table:** Demographic and clinical characteristics

		**Cohort 1 (n=96)**	**Cohort 2 (n=364)**	**Cohort 3 (n=508)**	**All patients (n=968)**
Age, years		35 (31–43)	36 (29–42)	35 (30–43)	35 (30–42)
Sex					
	Women	51 (53%)	218 (60%)	254 (50%)	523 (54%)
	Men	45 (47%)	146 (40%)	254 (50%)	445 (46%)
Positive WHO tuberculosis symptom screen		96 (100%)	329 (90%)	508 (100%)	933 (96%)
History of tuberculosis		52 (54%)	162 (45%)	225 (44%)	439 (45%)
Antiretroviral therapy		64 (67%)	153 (42%)	177 (35%)	394 (41%)
CD4 count, cells per μL		113 (40–262)	153 (53–313)	59 (23–122)	86 (33–190)
Diagnosis					
	Definite tuberculosis	47 (49%)	138 (38%)	415 (82%)	600 (62%)
	Possible tuberculosis	3 (3%)	37 (10%)	51 (10%)	91 (9%)
	Not tuberculosis	46 (48%)	189 (52%)	42 (8%)	277 (29%)
CD4 count, cells per μL					
	0–100	44 (46%)	135 (37%)	337 (66%)	516 (53%)
	101–200	19 (20%)	82 (23%)	115 (23%)	216 (22%)
	>200	30 (31%)	145 (40%)	56 (11%)	231 (24%)
	Unknown	3 (3%)	2 (1%)	0	5 (1%)
Outcome at 3 months					
	Died within 3 months	1 (1%)	19 (5%)	85 (17%)	105 (11%)
	Alive	58 (60%)	336 (92%)	416 (82%)	810 (84%)
	Lost to follow-up	0	9 (2%)	7 (1%)	16 (2%)
	No follow-up	37 (39%)	0	0	37 (4%)

Data are median (IQR), or n (%).

**Figure 3:**
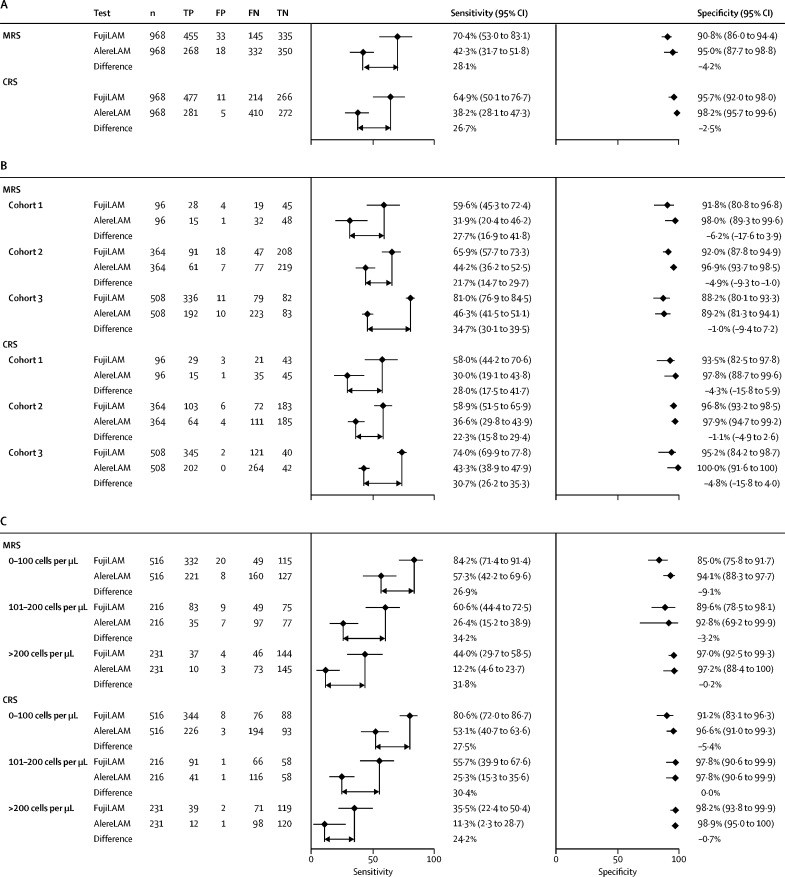
Sensitivity and specificity of FujiLAM versus AlereLAM against MRS and CRS Sensitivity and specificity of FujiLAM and AlereLAM assays for all cohorts combined (A), by cohort (B), and by CD4 count (C). Sensitivity and specificity estimates for (A) and (C) were based on analysis using a bivariate random-effects model. AlereLAM=Alere Determine TB LAM Ag assay. CRS=composite reference standard. FP=false positive. FN=false negative. FujiLAM=Fujifilm SILVAMP TB LAM assay. MRS=microbiological reference standard. TP=true positive. TN=true negative.

Overall, compared with the microbiological reference standard, the sensitivity of FujiLAM was 70·4% (95% CI 53·0–83·1) and 42·3% (31·7–51·8) for AlereLAM (difference 28·1%; figure 3). In comparison to the microbiological reference standard, the highest FujiLAM sensitivity was observed in cohort 3 (81·0% [76·9–84·5]), which enrolled patients with more advanced HIV-related immunosuppression (ie, more patients with a CD4 count less than 100 cells per μL) than cohort 2 (65·9% [57·7–73·3]) and cohort 1 (59·6% [45·3–72·4]). The sensitivity of both assays was higher in patients with lower CD4 counts. In patients with a CD4 count less than 100 cells per μL, FujiLAM had a sensitivity of 84·2% (71·4–91·4) compared with 57·3% (42·2–69·6) for AlereLAM (difference 26·9%). For patients with a CD4 count of more than 200 cells per μL sensitivity was 44·0% (29·7–58·5) for FujiLAM and 12·2% (4·6–23·7) for AlereLAM (difference 31·8%).

Using the composite reference standard, the sensitivity of both assays was slightly lower than that compared with the microbiological reference standard (64·9% [50·1–76·7] for FujiLAM *vs* 38·2% [28·1–47·3] for AlereLAM; difference 26·7%). Since the 95% CIs of the differences around sensitivity between FujiLAM and AlereLAM did not overlap, FujiLAM was considered to have significantly higher sensitivity than AlereLAM for all analyses, with the exception of cohort 1, in which the 95% CIs overlapped due to the small sample size (figure 3).

Compared with the microbiological reference standard, the specificity of FujiLAM was 90·8% (95% CI 86·0 to 94·4) and 95·0% (87·7 to 98·8) for AlereLAM, with no significant difference (−4·2%). Using the composite reference standard, overall estimates of specificity were increased to 95·7% (92·0 to 98·0) for FujiLAM and 98·2% (95·7 to 99·6) for AlereLAM, with no significant difference (−2·5%). Using the composite reference standard, specificity of the FujiLAM assay was lower among patients with CD4 counts equal to or less than 100 cells per μL (91·2%, 95% CI 83·1 to 96·3) than those with CD4 counts of 101–200 cells per μL (97·8% [95% CI 90·6 to 99·9]) and higher than 200 cells per μL (98·2% [93·8 to 99·9]; figure 3). Eight of the 11 FujiLAM false positive samples, using the composite reference standard, were from patients with CD4 counts equal to or less than 100 cells per μL. Additional information on the 11 FujiLAM false positive results is available in the appendix.

Using the composite reference standard, the positive predictive value for the three different cohorts ranged from 90·6–99·4% for FujiLAM and 93·8–100·0% for AlereLAM. The negative predictive value ranged from 24·8–71·8% for FujiLAM and 13·7–62·5% for AlereLAM. Positive likelihood ratios ranged from 8·9–18·5 for FujiLAM and 13·8–17·3 AlereLAM and negative likelihood ratios ranged from 0·3–0·4 for FujiLAM and 0·6–0·7 for AlereLAM (appendix).

All patients in cohort 2 (n=420) were eligible for the analysis of diagnostic yield (figure 2). Among the 420 eligible patients, only 153 (36%) could produce a sputum sample within the first 24 h of admission, whereas 418 (100%) were able to provide a urine sample, as described previously^11^ (appendix). 141 (34%) of 420 patients had microbiologically confirmed tuberculosis. 84 (60%) of 141 tuberculosis cases could be diagnosed with rapid tests using samples collected in the first 24 h of admission: 37 (26%) from sputum Xpert and 59 (42%) from urine Xpert using 1 mL urine. 57 (40%) of 141 tuberculosis diagnoses were not achieved in the first 24 h and were established by mycobacterial culture on any specimen collected at any point during patient admission, diagnosed by Xpert using concentrated samples from 20–40 mL urine or diagnosed by Xpert testing of specimens collected after the first 24 h. The additional specimens collected for culture and Xpert testing included ascitic fluid, blood, urine, sputum, cerebrospinal fluid, gastric lavage, pus, or pleural fluid (appendix).

**Figure 4:**
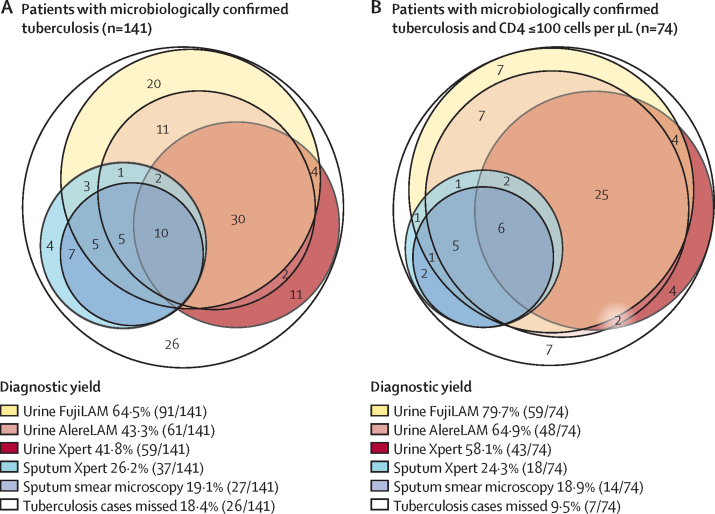
Diagnostic yields for cohort 2 Number of microbiologically confirmed tuberculosis diagnoses in all patients (A), and patients with CD4 counts of equal to or less than 100 cells per μL (B), detected by each diagnostic test on samples obtained within 24 h of hospital admission. Numbers represent the number of tuberculosis cases diagnosed by a given assay or assays. Tuberculosis cases missed includes diagnoses made by positive mycobacterial culture on any specimen collected at any point during patient admission or diagnoses made on the basis of Xpert testing of any specimen collected after the first 24 h of hospital admission. AlereLAM=Alere Determine TB LAM Ag assay. FujiLAM=Fujifilm SILVAMP TB LAM assay.

Figure 4 shows the diagnostic yield of FujiLAM and AlereLAM compared with other rapid diagnostic tests done within the first 24 h of hospital admission. 91 (65%) of 141 tuberculosis cases could have been diagnosed within a few hours of presentation with FujiLAM, compared with 61 (43%) of 141 cases with AlereLAM. A combination of sputum Xpert and FujiLAM within the first 24 h of admission would have been able to diagnose 102 (72%) of 141 microbiologically confirmed cases. A combination of sputum smear microscopy and FujiLAM would have yielded 98 (70%) of 141 diagnoses.

Overall, 18 (2%) of 1095 FujiLAM tests failed on the first attempt. Of the 15 tests that could be repeated, three failed on the second attempt resulting in a total overall error rate of 1·9% (21 of 1110 tests). FujiLAM failure rates are summarised in the appendix. The error rate for AlereLAM on the first attempt was 0·4% (four of 1095 tests) and all four repeat tests provided a result on the second attempt.

Inter-reader agreement was high for both FujiLAM and AlereLAM tests (appendix): 97·0% (938 of 967 reads; κ coefficient 0·94) for FujiLAM, and 96·7% (934 of 966 reads; κ coefficient 0·92) for AlereLAM.

## Discussion

In this study of 968 hospital inpatients with HIV in a high-burden setting, the FujiLAM point-of-care assay identified a significantly higher proportion of patients with tuberculosis than did the AlereLAM assay, while maintaining comparable specificity. In all sub-analyses, the sensitivity of FujiLAM was significantly higher (range 22–35%) than AlereLAM. FujiLAM had the highest sensitivity (84·2%) in patients with the highest risk of mortality (CD4 ≤100 cells per μL), which was 26·9% higher than that with AlereLAM. Combined with sputum Xpert, FujiLAM could diagnose nearly three-quarters of microbiologically confirmed tuberculosis within 24 h of hospital admission. The meta-analysis^4^, ^5^ that formed the basis of WHO recommendations for the use of AlereLAM reported an overall sensitivity of 45% in patients with HIV, which is similar to the AlereLAM sensitivity observed in this study (42%), suggesting that our study populations were similar to the populations included in the WHO meta-analysis. Collectively, these results suggest that, if implemented in clinical practice and linked with appropriate treatment, the FujiLAM point-of-care assay might be able to save lives by enabling earlier diagnosis of HIV-associated tuberculosis in a large proportion of hospital inpatients.^6^, ^20^, ^21^

The point estimates of FujiLAM specificity were lower than those for AlereLAM. Although the differences in specificity between FujiLAM and AlereLAM were not significant, the reduced specificity of both AlereLAM and FujiLAM could be partly explained by the use of an imperfect reference standard that lacks complete sensitivity. The existing reference standard is especially limited in its ability to identify tuberculosis in immuno-compromised patients with HIV,^22^ since these patients are more likely to have paucibacillary disease or extra-pulmonary tuberculosis than immunocompetent patients, making diagnosis more difficult. An imperfect reference standard could disproportionally affect a more sensitive test and result in increased false positives (ie, lower specificity with the more sensitive FujiLAM assay). The decreasing specificity observed with decreasing CD4 cell count in this study and the improved specificity observed with the composite reference standard in comparison to the microbiological reference standard further supports this explanation. Cross-reactivity of the antibodies used in FujiLAM to common urinary tract pathogens and fast-growing non-tuberculous mycobacteria has been excluded in previous studies.^7^

Our study has limitations that indicate further research is warranted. FujiLAM testing was done in a research laboratory setting using biobanked specimens collected from hospital inpatients. Although no technical reason exist as to why the test would perform differently in fresh versus frozen samples, this needs to be investigated. A previous study^23^ suggests that early morning urine collection could further improve the sensitivity of urinary lipoarabinomannan-based tuberculosis testing. This aspect could have important implications for clinical practice and should be addressed in future studies. FujiLAM has the potential to be implemented as a true point-of-care assay, but the feasibility of this approach and its effect on patient outcomes requires prospective assessment in relevant clinical settings. Both AlereLAM and FujiLAM cannot discern drug-resistant tuberculosis from drug-sensitive tuberculosis and therefore it is important that these rapid diagnostic tools are supplemented with sample collection for drug susceptibility testing.

Difficulties with regard to the assignment of diagnostic categories have been reported in previous literature^24^ and 10% of all eligible patients could not be classified in this study. The higher sensitivity of FujiLAM compared with AlereLAM was maintained when the unclassifiable group was included in a sensitivity analysis (appendix). 18 of the 121 unclassifiable patients had positive FujiLAM results and nine of these 18 patients died within 3 months of enrolment. Six of the nine FujiLAM positive patients who died were not started on tuberculosis treatment; assuming the assay had 100% specificity, these patients might have been true positives and thus might not have died if they were treated for tuberculosis.

The inclusion of patients from three cohorts from similar inpatient settings with different pretest probability of tuberculosis (appendix) provided an overview of the performance of FujiLAM in hospital inpatients with advanced HIV. However, the heterogeneity of these cohorts is also a limitation. As a result, we presented results per cohort and by CD4 strata. Grouped analysis with a Bayesian bivariate random-effects model was used to account for heterogeneity across the cohorts.

Renal tuberculosis infection has been proposed as the main cause of urinary lipoarabinomannan antigenuria and positive AlereLAM results.^25^ However, in this study, the more sensitive FujiLAM assay detected a number of urine Xpert-negative patients in whom renal tuberculosis is unlikely since Xpert would detect intact *M tuberculosis* bacteria in urine. This suggests that other mechanisms, such as passage of lipoarabinomannan or lipoarabinomannan fragments through the glomerular basement membrane, potentially exacerbated by HIV-associated nephropathy, might be more important than originally proposed. This is supported by findings from our previous study,^26^ which showed that blood and urine lipoarabinomannan concentrations correlate in patients with tuberculosis, and that of a 2018 study,^7^ which found that low urine lipoarabinomannan concentrations are detectable in immunocompetent HIV-negative patients with tuberculosis. The use of the more sensitive FujiLAM assay could help to increase the mechanistic understanding of how lipoarabinomannan enters urine.

The diagnostic yield analysis showed that only a minority of patients were able to produce sputum within the first 24 h of admission. Although this has been demonstrated in other studies of hospital inpatients,^27^, ^28^ the percentage of patients able to provide sputum was particularly low in cohort 2,^11^ despite substantial efforts to obtain the sample by a trained nurse, including access to sputum induction facilities. The inability to provide a sputum sample is likely a reflection of the severity of illness in this cohort and will be less pronounced in outpatients with HIV and tuberculosis symptoms, who also typically have higher CD4 cell counts.^29^ Studies of tuberculosis diagnostic assessments often exclude patients who cannot produce sputum, which can, particularly in the case of inpatients who are severely ill, lead to a biased study population and test assessment and exclude the population that would benefit most from a non-sputum based test.^30^

In conclusion, considering the higher sensitivity and rapid, point-of-care design of FujiLAM, this assay has the potential to transform the diagnosis of tuberculosis in hospital inpatients with HIV and, potentially, for people with HIV in the general population.

## Supplementary Materials

Supplementary appendix

Supplementary video The steps of the Fujifilm SILVAMP TB LAM test procedure to identify active tuberculosis from urine samples of patients with HIV.
