# Supplementary material for: Novel lipoarabinomannan point-of-care tuberculosis test for people with HIV: a diagnostic accuracy study
Source: Lancet Infect Dis. 2019 Aug;19(8):852–61. doi: 10.1016/S1473-3099(19)30001-5 (PMC6656794; doi:10.1016/S1473-3099(19)30001-5)
Supplement: Supplementary appendix [file mmc1.pdf]

THE LANCET Infectious Diseases

Supplementary webappendix

This webappendix formed part of the original submission and has been peer reviewed. We post it as supplied by the authors.

Supplement to: Broger T, Sossen B, du Toit E, et al. Novel lipoarabinomannan point-of-care tuberculosis test for people with HIV: a diagnostic accuracy study. *Lancet Infect Dis* 2019; published online May 30. http://dx.doi.org/10.1016/S1473-3099(19)30001-5.

Supplementary Appendix for:

Novel lipoarabinomannan point-of-care tuberculosis test for people living with HIV with superior sensitivity: a diagnostic accuracy study

Table of Contents

1) Table S1 – Study population, setting & location, eligibility, inclusion & exclusion criteria used for the three studies from which samples were included for testing 2
2) Table S2 – Specimen Collection and Testing Flow 3
3) Table S3 – Diagnostic Categories 6
4) Table S4 – Detailed Reasons for Exclusions and FujiLAM & AlereLAM results 7
5) Sensitivity analysis including the "unclassifiable category" 8
6) Heterogeneity statistic 10
7) Accuracy Estimates from Simple Pooling Compared to Accuracy Estimates from Analysis using the Bayesian bivariate random-effects model 11
8) Table S5 – Analysis of False Positive Fuji TB LAM Results 12
9) Table S6 – Positive Predictive Value (PPV), Negative Predictive Values (NPV), Positive Likelihood Ratio (LR+) and Negative Likelihood Ration (LR-) for the three cohorts 13
10) Figure S3 – Microbiologically Confirmed TB Diagnoses for Diagnostic Yield Analysis 14
11) Table S7 – FujiLAM Failure Rates and Errors 15
12) Table S8 – Agreement of Two Independent Test Readers 16
13) References 16

1) Table S1 – Study population, setting & location, eligibility, inclusion & exclusion criteria used for the three studies from which samples were included for testing

Cohort name	Cohort1	Cohort2	Cohort3
**Study population**	Adults suspected of having active TB disease	Unselected adults regardless of presenting symptoms	Adults suspected of having active TB disease
	High HIV prevalence setting	High HIV prevalence setting	High HIV prevalence setting
**Setting & location**	South Africa, Cape Town<br>-Khayelitsha Hospital<br>-Public sector district hospital that serves township communities<br>-Inpatient admissions	South Africa, Cape Town<br>-GF Jooste Hospital<br>-Public sector district hospital that served township communities<br>-Inpatients admissions	South Africa, Cape Town<br>-Khayelitsha Hospital<br>-Public sector district hospital that serves township communities<br>-Inpatient admissions
**Eligibility criteria**	Adults (≥18 years) presenting with symptomatic pulmonary disease thought to have TB	Unselected HIV infected adults (≥18 years) regardless of clinical presentation admitted to adult medical wards at Jooste Hospital	HIV infected adults (≥18 years) with CD4 counts ≤350 cells/µl and positive WHO symptom screen for TB
**Inclusion criteria**	-Informed consent from patient<br>-Suspected to have active TB based on clinical presentation<br>-Production of adequate quantity of sputum	-Informed consent from patient<br>-Regardless of presenting symptoms or reason for hospital admission<br>-HIV seropositive	-Informed consent as per study protocol<br>-Suspected to have active TB based on clinical presentation<br>-HIV seropositive<br>-CD4≤ 350 cells/µl
**Exclusion criteria**	-Participants receiving any anti-tuberculosis medication in the 60 days prior to enrolment<br>-Participants with only extra-pulmonary disease were excluded	-Patients that already received treatment for an existing diagnosis of TB at the time of admission	-3 or more doses of TB treatment received during the admission or has been on TB treatment within 1 month of admission<br>-Pregnant
**Enrolment period**	February 2016 – August 2017	June 2012 – October 2013	January 2014 – October 2016
**Participants considered for retrospective urinary LAM testing**	The full cohort (n=109) of the prospective cohort study. Patients with negative (n=28) or unknown (n=3) HIV status were excluded from the analysis.	The full cohort (n=420) of the prospective, consecutive cohort study. In comparison to the original study publication[1], where 427 patients were included, additional information was established leading to an additional seven patients being excluded due pre-existing TB at the time of enrollment.	The full cohort (n=659) of the prospective, observational cohort study.
**Study protocol, Principle Investigator and References**	Protocol: FIND Reference Materials Collection Protocol Version 14.0.<br>Principle Investigator Prof. Dr. Mark Nicol. Unpublished.	Protocol: "Systematic Screening for HIV associated Tuberculosis in Patients Admitted to GF Jooste District Hospital, Cape Town".<br>Principal Investigator Prof. Dr. Stephen D. Lawn. Published.[1]	Protocol: "Defining Interventions to reduce mortality in severe HIV-associated Tuberculosis" Version 4.0.<br>Principal Investigator Prof. Dr. Graeme Meintjes. Unpublished.

2) Table S2 – Specimen Collection and Testing Flow

Cohort 1* (109 patients)				
**Time point**	**Clinical**	**Urine**	**Blood**	**Sputum**
**Enrollment Day1**	· Informed consent<br>· Inclusion and Exclusion<br>· Clinical questionnaire	**Pooled Urine (Urine1 and Urine2)**<br>·Sample collection for retrospective testing (Fuji LAM and Alere LAM)<br>· Urinary Xpert (30-40 ml)<br>· Urinary Dipstick<br>· Alere LAM (fresh)	**Venous Blood**<br>·Blood culture (BACTEC Myco F/Lytic)<br>·HIV testing (RDT)<br>·CD4 cell count (Flow Cytometry)	**Sputum 1 and Sputum 2**<br>·Smear Microscopy<br>·Xpert<br>·Solid Culture (LJ) /Speciation<br>·Liquid Culture (MGIT) /SpeciationMGIT<br>·DST
**Enrollment Day2**		**Urine 3**<br>· Urinary Dipstick<br>· Alere LAM (fresh)		**Sputum 3**<br>·Smear Microscopy<br>·Xpert<br>·Solid Culture (LJ) /Speciation<br>·Liquid Culture (MGIT) /SpeciationMGIT<br>·DST
**Follow-up (8 weeks)**	· Clinical questionnaire			**Sputum 4**<br>·Smear Microscopy<br>·Xpert<br>·Solid Culture (LJ) /Speciation<br>·Liquid Culture (MGIT) /SpeciationMGIT<br>·DST
**Total no. of samples collected (702 in total)**		**211**	**107**	**384**
**Average no. of samples/patient (6.4/patient)**		**1.9**	**1.0**	**3.5**
**No (%) of patients with ≥1 sample collected**		**107 (98.1%)**	**107 (98.1%)**	**107 (98.1%)**
**Total no of culture and Xpert done (1479 in total)**		**107**	**107**	**1265**
**Average number of culture+Xpert/patient (13.6/patient)**		**1.0**	**1.0**	**11.6**

Cohort 2* (420 patients)					
**Time point**	**Clinical**	**Urine**	**Blood**	**Sputum**	**Other specimen**
**Enrollment Day1**	· Informed consent<br>· Inclusion and Exclusion<br>· Clinical questionnaire	**Urine 1**<br>·Sample collection for retrospective testing (Fuji LAM and Alere LAM)<br>· Urinary Xpert (2 ml)<br>· Urinary Xpert (30-40 ml)<br>· Urinary Culture	**Venous Blood**<br>·Blood culture (BACTEC Myco F/Lytic)<br>·HIV testing (RDT)<br>·CD4 cell count (Flow Cytometry)	**Sputum 1 and Sputum 2 (sputum induction if necessary)**<br>·Smear Microscopy<br>·Xpert<br>·Solid Culture (LJ) /Speciation<br>·Liquid Culture (MGIT) /SpeciationMGIT<br>·DST	
**Throughout admission period, if available (including samples from routine)**	· Clinical information		**Venous Blood**<br>·Blood culture (BACTEC Myco F/Lytic)	**Sputum(s)**<br>·Xpert<br>·Culture	**Other non-respiratory samples** for Xpert or culture including (Ascitic fluid, bone marrow, cerebrospinal fluid, fine needle aspirate, gastric lavage, pus, pleural fluid, stool)
**Follow-up (12 weeks)**	· Clinical information				
**Total no. of samples collected (1,745 in total)**		**418**	**469**	**615**	**243**
**Average no. of samples/patient (4.2/patient)**		**1.0**	**1.1**	**1.5**	**0.6**
**No. (%) of patients with ≥1 sample collected in the first 24 hours**		**418 (99.5%)**	**0 (0%)**	**153 (36.4%)**	**0 (0%)**
**No (%) of patients with ≥1 sample collected during admission**		**418 (99.5%)**	**410 (97.6)**	**245 (58.3%)**	**177 (41.5%)**
**Total no. of culture and Xpert done (2391 in total)**		**833**	**469**	**871**	**218**
**Average number of culture+Xpert/patient (5.7/patient)**		**2.0**	**1.1**	**2.1**	**0.5**

Cohort 3* (508 patients)					
**Time point**	**Clinical**	**Urine**	**Blood**	**Sputum**	**Other specimen**
**Before the 3rd dose of TB treatment**	· Informed consent (as per protocol)<br>· Inclusion and Exclusion<br>· Clinical questionnaire<br>· Clinical examination	· Sample collection for retrospective testing (Fuji LAM and Alere LAM)<br>· Urinary Xpert (30-40 ml)	**Venous Blood**<br>·Blood culture (BACTEC Myco F/Lytic)<br>·GenoType MTBDRplus assay (Hain Lifesciences) on positive cultures<br>·DST<br>·HIV viral load<br>·CD4 cell count	**Sputum induction performed if necessary**<br>·Smear Microscopy<br>·Xpert<br>·Liquid Culture (MGIT)<br>·GenoType MTBDRplus assay (Hain Lifesciences) on positive cultures<br>·DST	
**Throughout admission period, if available (including samples from routine)**	· Clinical information	**Urine**<br>·Culture	**Venous Blood**<br>·Blood culture (BACTEC Myco F/Lytic)	**Sputum(s)**<br>·Xpert<br>·Culture	**Other non-respiratory samples** for Xpert or culture including ascitic fluid, cerebrospinal fluid, fine needle aspirate of nodes, puss, pleural fluid and pericardial fluid.
**Follow-up (12 weeks)**	· Clinical information				

**Total no. of culture and Xpert done (2527 in total)**		**559**	**633**	**1125**	**210**
**Average no. of culture+Xpert/patient (5.0/patient)**		**1.1**	**1.2**	**2.2**	**0.4**

*Cohort1 was designed to systematically collect samples in the first two days and at follow-up. Cohort2 was designed to systematically collect samples within 24 hours of admission. Cohort3 routinely collected samples before the 3rd dose of TB treatment. Both, Cohort2 and Cohort3, allowed for additional clinical samples during the hospital admission to contribute towards reference standard and number of samples collected are indicated.

3) Table S3 – Diagnostic Categories

Category	Cohort1	Cohort2	Cohort3
**Definite TB**	**Any culture or any Xpert (baseline) positive for MTB**<br>≥1 Positive culture (solid, liquid, sputum or blood) & confirmed MTB complex at baseline (Cross-contamination: A single LJ culture with ≤ 20 colonies or a single MGIT culture with MTB growth ≥28 days per patient are excluded from analysis)<br>OR<br>≥1 Positive Xpert (sputum or urine) at baseline	**Any culture or any Xpert positive for MTB from any anatomic sample**<br>≥1 Positive culture (solid, liquid) & confirmed MTB complex from any clinical sample during enrolment admission<br>OR<br>≥1 Positive Xpert (sputum, urine or any other clinical sample during enrolment admission	**Any culture or any Xpert positive for MTB**<br>≥1 Positive culture (solid, liquid) & confirmed MTB complex from any clinical sample during enrolment admission<br>OR<br>≥1 Positive Xpert (sputum or urine or any other clinical sample) during enrolment admission
**Possible TB**	**Any patient not meeting definite TB or Not TB classification who is started on TB treatment or has positive laboratory findings on follow-up**<br>Empiric TB treatment started by the healthcare provider<br>Or<br>Positive sputum culture and/or sputum Xpert and/or sputum smear on follow-up	Any patient not meeting definite TB or Not TB classification, who either has clinical/radiographic features suggestive of TB<br>OR<br>Any patient who is started on TB treatment	Any patient not meeting definite TB or Not TB classification who is started on TB treatment and TB treatment not stopped due to alternative diagnosis<br>AND<br>Alive at 12 weeks, symptoms improved on TB treatment
**Not TB**	**All microscopy, culture and Xpert tests negative for MTB, not started on TB treatment, recovers and has negative follow-up tests**<br>All culture negative (sputum, blood, incl. follow-up, with at least 2 LJ or MGIT with no culture growth after >56 days and >42 days)<br>AND<br>At least 2 valid negative culture or Xpert results from 2 or more independent samples obtained from 2 or more different anatomic sites, such as blood, sputum, or urine<br>AND<br>All Xpert negative (incl. follow-up)<br>AND<br>All smear microscopy results negative (incl. follow-up)<br>AND<br>Treatment not initiated by healthcare providers<br>AND<br>Improvement or full recovery at 8-week follow-up in the absence of TB treatment	All microscopy, cultures and Xpert tests negative for MTB, not started on TB treatment during hospitalization, and alive at 90 days<br>AND<br>At least 2 valid (and negative) culture or Xpert results available (at least 1 of which was a culture result)	All microscopy, cultures and Xpert tests negative for MTB, not started on TB treatment, alive at 12 weeks and not on TB treatment at 12 weeks.<br>AND<br>At least 1 valid (and negative) culture result available
**Unclassifiable**	**All participants that do not fall into groups "Definite TB", "Not TB" or "Possible TB"**<br>i.e.:<br>No symptom resolution on follow-up (same or worse) for baseline negative participants<br>Or<br>Loss to follow-up for baseline negative participants<br>Or<br>Passed away for baseline negative participants<br>Or<br>Insufficient laboratory results (i.e. participants with <2 valid cultures)<br>OR<br>Baseline smear microscopy positive but culture and Xpert negative	**All participants that do not fall into group "Definite TB", "Not TB" or "Possible TB"**<br>i.e.:<br>Participants without definite or probable TB but died within 90 days<br>OR<br>Participants without definite or probable TB but were LTFU within 90 days<br>OR<br>Patient with baseline smear microscopy positive but culture and Xpert negative and not falling into the "possible TB" category (ie, not started on TB treatment)	**All participants that do not fall into group "Definite TB", "Not TB" or "Possible TB"**<br>i.e.:<br>Without definite TB but started TB treatment, while still on TB treatment, deteriorated/died/lost to follow-up or the symptoms not improved at 12 weeks.<br>OR<br>Loss to follow-up for baseline negative participants<br>OR<br>Died before start of TB treatment for baseline negative participants<br>OR<br>Insufficient laboratory results (i.e. participants with no valid cultures)<br>OR<br>Baseline smear microscopy positive but culture and Xpert negative and not falling into the "possible TB" category<br>OR<br>All microscopy, cultures and Xpert tests negative for MTB, started on TB treatment and TB treatment stopped during the same admission due to alternative diagnosis being made

4) Table S4 – Detailed Reasons for Exclusions and FujiLAM & AlereLAM results

Reason for Exclusion	Cohort1	Cohort2	Cohort3	All Cohorts	All Cohorts FujiLAM+	All Cohorts AlereLAM+
	no.	no.	no.	no.	no. (%)	no. (%)
**Unclassifiable**	10	46	65	121	18 (15%)	12 (10%)
BL-, no Rx, died	3	31	9	43	6 (14%)	5 (12%)
BL-, Rx, died	0	0	19	19	3 (16%)	2 (11%)
BL-, no Rx, LTFU	1	14	2	17	2 (12%)	1 (6%)
BL-, Rx, alternative diag., Rx stopped	0	0	9	9	2 (22%)	1 (11%)
BL-, Rx, deteriorated	0	0	7	7	0 (0%)	0 (0%)
BL-, Rx, symptoms not improved	0	0	4	4	1 (25%)	0 (0%)
BL-, no Rx, symptoms not improved	4	0	0	4	0 (0%)	0 (0%)
No valid culture/Xpert results	0	0	3	3	1 (33%)	0 (0%)
BL-, Rx, LTFU	0	0	11	11	3 (27%)	3 (27%)
BL-, no Rx initiated, but Rx initiated later	0	0	1	1	0 (0%)	0 (0%)
Smear+, Culture-	2	1	0	3	0 (0%)	0 (0%)
**No urine sample**	3	9	81	93		
**Missing index-test result**	0	1	5	6		
**TOTAL Excluded**	**13**	**56**	**151**	**220**		

no., number of patients
BL-, baseline TB negative
Rx, anti-tuberculosis treatment
FU, follow-up
LTFU, lost to follow-up
Smear+, sputum smear microscopy positive,
Culture-, mycobacterial culture negative and
Diag., diagnosis

5) Sensitivity analysis including the "unclassifiable category"

121 patients were grouped as "unclassifiable" (Figure 2), as per pre-specified diagnostic categories (Table S3). To assess the impact of these study exclusions on accuracy estimates, we performed a sensitivity analysis by including the "unclassifiable" category in the microbiological reference standard (MRS) and composite reference standard (CRS), as shown in the modified flow diagram (Figure S1, below and Figure 2 for the original flow diagram). The sensitivity analysis was performed using the same statistical methods as the original analysis. As shown in Figure S2, inclusion of the "unclassifiable" group led to a decrease in specificity of 1.0% and 1.0% for FujiLAM and AlereLAM respectively against the MRS compared to the original analysis. Sensitivity decreased 8.9% and 4.9% for FujiLAM and AlereLAM respectively against the CRS compared to the original analysis. Although the sensitivity difference between FujiLAM and AlereLAM decreased by 4.1%, FujiLAM sensitivity was still 22.6% higher and superior to AlereLAM sensitivity.

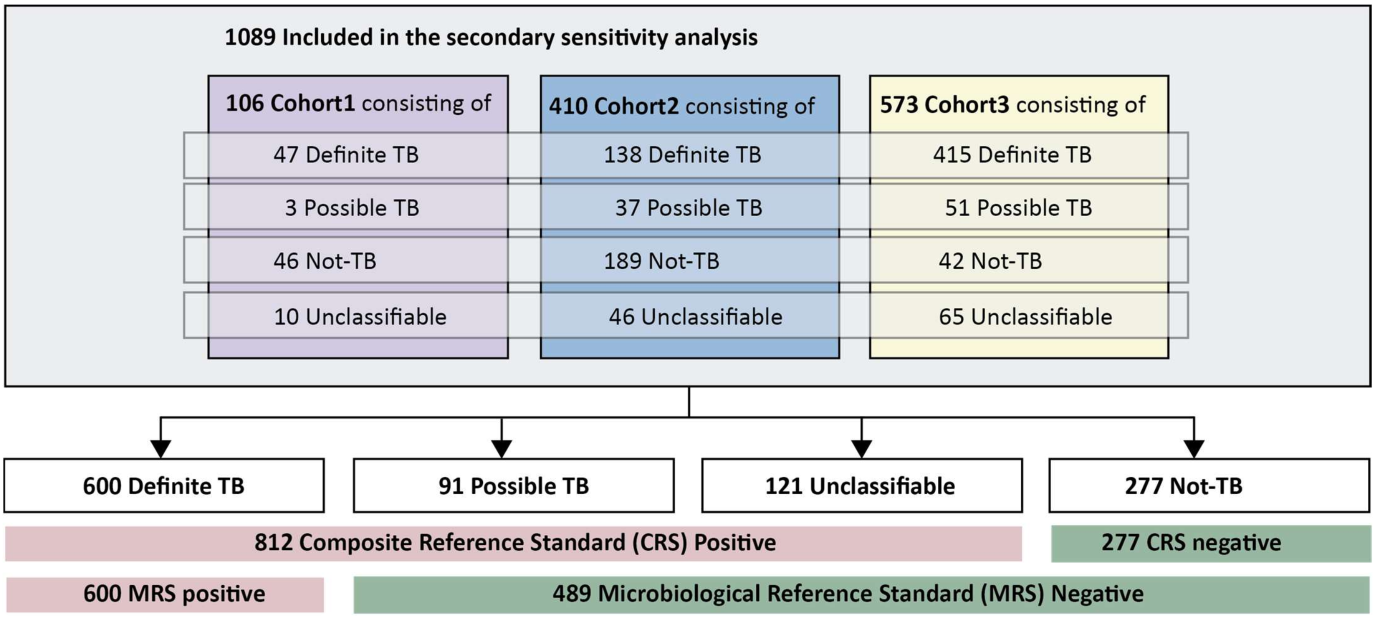

Figure S1: Modified flow diagram that includes the "unclassifiable" category for sensitivity analysis.

MRS

(A) Original analysis

MRS positive (Definite TB) / MRS negative (Possible TB and Not TB)

	Test	N	TP	FP	FN	TN	Sensitivity [95% CI]		Specificity [95% CI]	
All HIV+ MRS	FujiLAM	968	455	33	145	335	70.4%	[53.0 - 83.1]	90.8%	[86.0 - 94.4]
	AlereLAM	968	268	18	332	350	42.3%	[31.7 - 51.8]	95.0%	[87.7 - 98.8]
	ΔSn and ΔSp						28.1%		-4.1%	

(B) Sensitivity analysis

MRS positive (Definite TB) / MRS negative (Possible TB + Not TB + Unclassifiable)

	Test	N	TP	FP	FN	TN	Sensitivity [95% CI]		Specificity [95% CI]	
All HIV+ MRS	FujiLAM	1089	455	51	145	438	70.4%	[53.0 - 83.1]	89.8%	[84.3 - 94.1]
	AlereLAM	1089	268	30	332	459	42.3%	[31.7 - 51.8]	94.0%	[86.9 - 98.1]
	ΔSn and ΔSp						28.1%		-4.2%	

Difference (B) - (A)	Difference FujiLAM Specificity	-1.0%
	Difference AlereLAM Specificity	-1.0%
	Difference ΔSp	-0.1%

CRS

(A) Original analysis

CRS positive (Definite TB+Possible TB) / CRS negative (Not TB)

	Test	N	TP	FP	FN	TN	Sensitivity [95% CI]		Specificity [95% CI]	
All HIV+ CRS	FujiLAM	968	477	11	214	266	64.9%	[50.1 - 76.7]	95.7%	[92.0 - 98.0]
	AlereLAM	968	281	5	410	272	38.2%	[28.1 - 47.3]	98.2%	[95.7 - 99.6]
	ΔSn and ΔSp						26.7%		-2.4%	

(B) Sensitivity analysis

CRS positive (Definite TB+Possible TB+Unclassifiable) / CRS negative (Not TB)

	Test	N	TP	FP	FN	TN	Sensitivity [95% CI]		Specificity [95% CI]	
All HIV+ CRS	FujiLAM	1089	495	11	317	266	56.0%	[40.0 - 70.1]	95.7%	[92.0 - 98.0]
	AlereLAM	1089	293	5	519	272	33.3%	[23.5 - 42.8]	98.2%	[95.7 - 99.6]
	ΔSn and ΔSp						22.6%		-2.4%	

Difference (B) - (A)	Difference FujiLAM Sensitivity	-8.9%
	Difference AlereLAM Sensitivity	-4.9%
	Difference ΔSn	-4.1%

Figure S2: Diagnostic accuracy from the original meta-analysis (A) compared to the results of the sensitivity analysis (B) for FujiLAM and AlereLAM in PLHIV.

6) Heterogeneity statistic

Heterogeneity was assessed for pooled sensitivity estimates using Cochran's Q-test and is presented in the table below. As expected there was significant heterogeneity for the overall pooled analyses, which led to the decision to use the Bayesian bivariate random-effects model to account for study design differences. In CD4 strata, Cochran's Q-test failed to show significant heterogeneity in all except one CD4 strata suggesting that different levels of immunosuppression explain the differences between cohorts and that CD4-based subgroup analyses can remove heterogeneity. Nevertheless for reasons of consistency the (relatively conservative) Bayesian bivariate random-effects mode were also used for the CD4-based subgroup analyses.

MRS	Test	N	TP	FP	FN	TN	Sensitivity	[95% CI]	Q	p-value	
All HIV+	FujiLAM	968	455	33	145	335	70.4%	[53.0 - 83.1]	17.7	0.0002	*
	AlereLAM	968	268	18	332	350	42.3%	[31.7 - 51.8]	4.0	0.1387	

CRS	Test	N	TP	FP	FN	TN	Sensitivity	[95% CI]	Q	p-value	
All HIV+	FujiLAM	968	477	11	214	266	64.9%	[50.1 - 76.7]	15.9	0.0004	*
	AlereLAM	968	281	5	410	272	38.2%	[28.1 - 47.3]	5.3	0.0697	

MRS	Test	N	TP	FP	FN	TN	Sensitivity	[95% CI]	Q	p-value	
0 – 100 c/µl	FujiLAM	516	332	20	49	115	84.2%	[71.4 - 91.4]	5.1	0.0779	
	AlereLAM	516	221	8	160	127	57.3%	[42.2 - 69.6]	5.2	0.0752	
101 – 200 c/µl	FujiLAM	216	83	9	49	75	60.6%	[44.4 - 72.5]	2.6	0.2719	
	AlereLAM	216	35	7	97	77	26.4%	[15.2 - 38.9]	2.2	0.3261	
>200 c/µl	FujiLAM	231	37	4	46	144	44.0%	[29.7 - 58.5]	2.3	0.3123	
	AlereLAM	231	10	3	73	145	12.2%	[4.6 - 23.7]	4.8	0.0904	

CRS	Test	N	TP	FP	FN	TN	Sensitivity	[95% CI]	Q	p-value	
0 – 100 c/µl	FujiLAM	516	344	8	76	88	80.6%	[72.0 - 86.7]	1.7	0.4301	
	AlereLAM	516	226	3	194	93	53.1%	[40.7 - 63.6]	3.5	0.1752	
101 – 200 c/µl	FujiLAM	216	91	1	66	58	55.7%	[39.9 - 67.6]	3.0	0.2224	
	AlereLAM	216	41	1	116	58	25.3%	[15.3 - 35.6]	2.1	0.3566	
>200 c/µl	FujiLAM	231	39	2	71	119	35.5%	[22.4 - 50.4]	3.9	0.1454	
	AlereLAM	231	12	1	98	120	11.3%	[2.3 - 28.7]	8.0	0.0185	*

*significant

7) Accuracy Estimates from Simple Pooling Compared to Accuracy Estimates from Analysis using the Bayesian bivariate random-effects model

Meta-analysis (MRS, All HIV+)

MRS	Test	N	TP	FP	FN	TN	Sensitivity [95% CI]*	Specificity [95% CI]*
All HIV+	FujiLAM	968	455	33	145	335	70.4% [53.0 - 83.1]	90.8% [86.0 - 94.4]
	AlereLAM	968	268	18	332	350	42.3% [31.7 - 51.8]	95.0% [87.7 - 98.8]
	ΔSn and ΔSp						28.1%	-4.2%

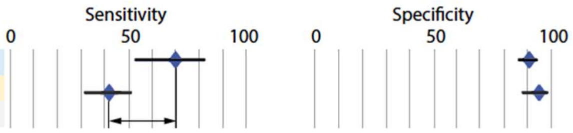

Simple Pooling (MRS, All HIV+)

MRS	Test	N	TP	FP	FN	TN	Sensitivity [95% CI]	Specificity [95% CI]
All HIV+	FujiLAM	968	455	33	145	335	75.8% [72.3,79.1]	91.0% [87.7,93.5]
	AlereLAM	968	268	18	332	350	44.7% [40.7,48.7]	95.1% [92.4,96.9]
	ΔSn and ΔSp						31.2% [27.4,35.1]	-4.1% [-7.6,-0.8]

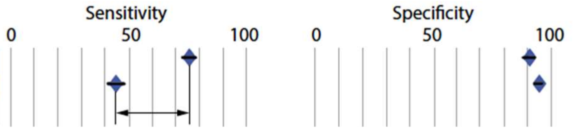

Meta-analysis (CRS, All HIV+)

CRS	Test	N	TP	FP	FN	TN	Sensitivity	Specificity
All HIV+	FujiLAM	968	477	11	214	266	64.9% [50.1 - 76.7]	95.7% [92.0 - 98.0]
	AlereLAM	968	281	5	410	272	38.2% [28.1 - 47.3]	98.2% [95.7 - 99.6]
	ΔSn and ΔSp						26.7%	-2.5%

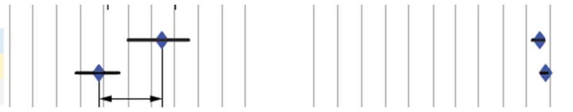

Simple Pooling (CRS, All HIV+)

CRS	Test	N	TP	FP	FN	TN	Sensitivity	Specificity
All HIV+	FujiLAM	968	477	11	214	266	69.0% [65.5,72.4]	96.0% [93.0,97.8]
	AlereLAM	968	281	5	410	272	40.7% [37.1,44.4]	98.2% [95.8,99.2]
	ΔSn and ΔSp						28.4% [24.8,32.1]	-2.2% [-5.4,0.7]

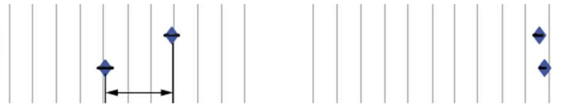

8) Table S5 – Analysis of False Positive Fuji TB LAM Results (Abbreviations; UK, Unknown, CRP, C-reactive protein; CVA, Cerebrovascular accident ART, antiretroviral therapy; NTM, Nontuberculous mycobacteria; CAP, community acquired pneumonia; CKD, chronic kidney disease; Hb, haemoglobin; LRTI, lower respiratory tract infection; TB, tuberculosis)

Cohort	Age (years)	Sex	CD4 (cells/µl)	WHO symptom screen	TB History (time prior to enrollment)	CRP (mg/l)	Hb (g/dl)	Sputum *M.tb* Xpert and Culture	Blood *M.tb* Culture	Urine *M.tb* Xpert	Clinical follow-up (8-12 weeks)	ART	Alternate diagnosis and clinical information
1	29	Female	21	Positive	yes (~4 months)	UK	UK	Xpert negative Culture negative	Negative	Negative	Alive improved	yes	none
1	28	Male	12	Positive	no	UK	UK	Xpert negative Culture negative	Negative	Negative	Alive improved	no	none
1	46	Male	2	Positive	no	UK	UK	Xpert negative Culture negative	Negative	Negative	Alive improved	no	none
2	39	Female	136	Positive	yes (~1 year)	9.2	9	Could not provide sputum	Negative	Negative	Alive	yes	TB not suspected, acute illness. Patient presents with acute stroke (R-sided CVA)
2	50	Male	49	Positive	no	141	7	Xpert negative	Negative	Negative	Alive	naïve	Evidence of NTM on 2 sputum cultures. TB clinically suspected and presented with acute illness, CKD 2. Had Streptococcus pneumoniae in blood and Pneumonia-like picture
2	47	Male	40	Positive	yes (time unknown)	338	10.9	Xpert negative Culture negative	Negative	Negative	Alive	interrupted	TB clinically suspected and presented with acute illness. Clinically felt to be CAP. Diagnosis: Right lobar consolidation
2	58	Male	52	Positive	yes (~1 year)	112	7.7	Xpert negative Culture negative	Negative	Negative	Alive	interrupted	TB clinically suspected with chronic illness. Illness felt to be likely LRTI, although TB in differential diagnosis
2	41	Female	406	Positive	yes (~2 years)	7	13.8	Could not provide sputum	Negative	Negative	Alive	interrupted	Interrupted ART. TB not clinically suspected, subacute illness. CKD 3. Admission due to psychosis.
2	35	Male	252	Positive	yes (~2 years)	2	14	could not provide sputum	Negative	Negative	Alive	interrupted	Interrupted ART. TB not clinically suspected, acute illness. Likely neurocysticercosis.
3	28	Female	10	Positive	yes (time unknown)	241	7.9	Xpert negative Culture negative	not done	Negative	Deteriorated	yes	*E.coli* sepsis (blood and urine cult). Completed 5 days treatment and was discharged. During follow up, treated for CAP which did not require admission
3	31	Male	44	Positive	yes (time unknown)	261	9.8	Xpert negative Culture negative	Negative	Negative	Alive improved	UK	CAP

9) Table S6 – Positive Predictive Value (PPV), Negative Predictive Values (NPV), Positive Likelihood Ratio (LR+) and Negative Likelihood Ration (LR-) for the three cohorts

MRS	Test	N	TP	FP	FN	TN	Prevalence	PPV	NPV	LR+	LR-
Cohort1	FujiLAM	96	28	4	19	45	49.0%	87.5%	70.3%	7.3	0.4
	AlereLAM	96	15	1	32	48	49.0%	93.8%	60.0%	15.6	0.7
	ΔPPV and ΔNPV							-6.3%	10.3%		
Cohort2	FujiLAM	364	91	18	47	208	37.9%	83.5%	81.6%	8.3	0.4
	AlereLAM	364	61	7	77	219	37.9%	89.7%	74.0%	14.3	0.6
	ΔPPV and ΔNPV							-6.2%	7.6%		
Cohort3	FujiLAM	508	336	11	79	82	81.7%	96.8%	50.9%	6.8	0.2
	AlereLAM	508	192	10	223	83	81.7%	95.0%	27.1%	4.3	0.6
	ΔPPV and ΔNPV							1.8%	23.8%		

CRS	Test	N	TP	FP	FN	TN					
Cohort1	FujiLAM	96	29	3	21	43	52.1%	90.6%	67.2%	8.9	0.4
	AlereLAM	96	15	1	35	45	52.1%	93.8%	56.3%	13.8	0.7
	ΔPPV and ΔNPV							-3.1%	10.9%		
Cohort2	FujiLAM	364	103	6	72	183	48.1%	94.5%	71.8%	18.5	0.4
	AlereLAM	364	64	4	111	185	48.1%	94.1%	62.5%	17.3	0.6
	ΔPPV and ΔNPV							0.4%	9.3%		
Cohort3	FujiLAM	508	345	2	121	40	91.7%	99.4%	24.8%	15.5	0.3
	AlereLAM	508	202	0	264	42	91.7%	100.0%	13.7%	ND*	0.6
	ΔPPV and ΔNPV							-0.6%	11.1%		

*ND; Not defined

10) Figure S3 – Microbiologically Confirmed TB Diagnoses for Diagnostic Yield Analysis

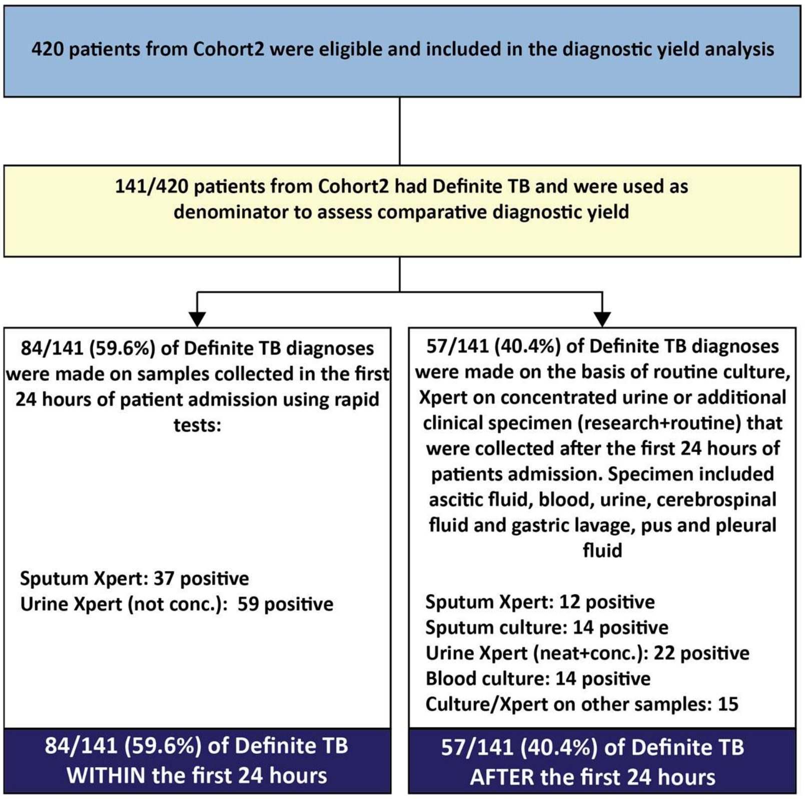

11) Table S7 – FujiLAM Failure Rates and Errors

**Normal runs, first attempt**	**1095**	**100.0%**
**Failure on 1st try, no interpretation possible**	**18**	**1.6%**
No control line, repeated	7	0.6%
User error, repeated	3	0.3%
Shadow in reading window, repeated	1	0.1%
Shadow on reading window, not repeated by lab staff, excluded from analysis*	3	0.3%
Liquid did not move, repeated	2	0.2%
Button 3 failure, repeated	1	0.1%
Liquid run back, repeated	1	0.1%
**Repeat runs, second attempt**	**15**	**100.0%**
**Failure on repeat, no interpretation possible**	**3**	**20.0%**
No control line, not repeated, excluded from analysis*	2	13.3%
Liquid did not move, not repeated, excluded from analysis*	1	6.7%

* FujiLAM errors led to the exclusion of 6 patients from the analysis

**Total tests run**	**1110**	**100.0%**
Normal runs	**1095**	98.6%
Repeats	**15**	1.4%
**Total failures (1st try + repeats)**	**21**	**1.9%**
Failure on 1st try, no interpretation possible	18	1.6%
Failure on repeat, no interpretation possible	3	0.3%

12) Table S8 – Agreement of Two Independent Test Readers

FujiLAM all tests

N=967*	R2+	R2-
R1+	473	11
R1-	18	465

p0	0.970	
kappa	0.940	[0.92-0.96]

AlereLAM all tests

N=966*	R2+	R2-
R1+	270	18
R1-	14	664

p0	0.967	
kappa	0.921	[0.89-0.95]

*Tests with a valid final call (either positive or negative), where one of the readers judged the test as Indeterminate were excluded from the agreement analysis (1 in the case of FujiLAM and 2 in the case of AlereLAM).

13) References

1. Lawn SD, Kerkhoff AD, Burton R, Schutz C, Boulle A, Vogt M, et al. Diagnostic accuracy, incremental yield and prognostic value of Determine TB-LAM for routine diagnostic testing for tuberculosis in HIV-infected patients requiring acute hospital admission in South Africa: a prospective cohort. BMC Med. 2017;15(1):67.
